# Dry Eye Disease: Consideration for Women's Health

**DOI:** 10.1089/jwh.2018.7041

**Published:** 2019-04-10

**Authors:** Cynthia Matossian, Marguerite McDonald, Kendall E. Donaldson, Kelly K. Nichols, Sarah MacIver, Preeya K. Gupta

**Affiliations:** ^1^ Matossian Eye Associates, Pennington and Pennington, New Jersey.; ^2^ Ophthalmic Consultants of Long Island, Lynbrook, New York.; ^3^ Department of Ophthalmology, Bascom Palmer Eye Institute, University of Miami Miller School of Medicine, Miami, Florida.; ^4^ School of Optometry, University of Alabama at Birmingham, Birmingham, Alabama.; ^5^ School of Optometry and Vision Science, University of Waterloo, Ontario, Canada.; ^6^ Division of Cornea and Refractive Surgery, Department of Ophthalmology, Duke University Eye Center, Durham, North Carolina.

**Keywords:** epidemiology, postmenopausal women, primary care, dry eye disease, autoimmune disease, comorbid conditions, quality of life, chronic, progressive

## Abstract

Dry eye disease (DED) is a multifactorial disorder of the ocular surface and tear homeostasis that can result in discomfort, pain, and visual disturbance. Untreated, DED can become chronic, progressive, and significantly affect an individual's quality of life. Women are disproportionately affected by DED, are diagnosed at a younger age, and experience more severe symptoms compared with men. DED is associated with a wide range of comorbid conditions; there is a strong association between DED and autoimmune disorders, especially those that affect women at many times the rate of men. Treatment response questionnaires indicate women respond better to a wellness model of treatment for DED than men. Furthermore, women's health care-seeking behaviors provide opportunities for general practitioners, specialists, and women's health centers to help identify women with DED or at risk for DED for referral to an eye care specialist. This review of the prevalence of DED in women, and gender and sex-specific aspects of DED, highlight a significant opportunity for action. Earlier diagnosis and treatment of this common but burdensome condition could significantly improve a woman's quality of life.

## Introduction

Dry eye disease (DED) is an ocular disorder that is often characterized by symptoms of eye dryness, discomfort, and sensitivity to light.^1,2^ It is highly prevalent, increases with age and, as is the case with many ocular conditions, disproportionately affects women.^3–11^ Untreated, DED may become chronic and progressive and can significantly affect one's quality of life.^7,12–18^

Greater utilization of the health care system by women provides the opportunity for DED to be recognized and diagnosed earlier.^12,19^ Furthermore, women's health care settings, which are designed to offer “one-stop shopping” for medical needs compared with general internal medicine practices, and primarily utilized by younger individuals,^20^ may provide additional opportunities to identify, study, educate, and treat women with DED or at risk of developing DED. However, the literature on DED currently lacks a comprehensive review of the interrelationship of DED with women's health. Such a review could be used by clinicians outside of eye care, in addition to ophthalmologists and optometrists, to identify women with DED, or at higher risk of developing DED.

DED is a multifactorial disease, with a highly variable presentation in both clinical signs and patient-reported symptoms, making it difficult for clinicians outside of eye care to recognize.^21–23^ There are many symptoms other than “dryness” that are associated with dry eye that clinicians might not know, for example, blurred vision. However, a growing body of research investigating the root pathology of DED, namely dysregulation of the ocular surface, including the ocular surface immune response, has led to greater understanding of the risk factors associated with DED.^10,24–31^ With these advances, it may be easier for non-eye care professionals to identify possible DED from risk factors, such as chronic illnesses and autoimmune diseases, combined with symptoms of ocular discomfort or visual disturbance.

We performed a nonsystematic literature search on topics focusing on the pathophysiology of DED and key epidemiology studies that have assessed differences in DED prevalence. Published studies discussed in this review include data according to self-reported gender (woman or man; *e.g.*, surveys) or biological sex (female or male; *e.g.*, hormonal studies). We explore the biological sex and hormonal differences in ocular structure, functioning, and health that contribute to the higher DED prevalence in women. We recapitulate the presenting signs and symptoms of DED that differ between women and men, and summarize some of the current research on DED-associated comorbidities that are more prevalent in, or specific to, women.

There is a high prevalence of DED in several Asian populations.^6^ However, when the data are stratified by gender, distribution of the disease is similar to studies in primarily Caucasian populations irrespective of ethnicity.^32^ In this review, we do not discuss the relationship between ethnicity and DED.

It is our hope that this review reaches a broad audience of health care professionals, with the goal of contributing to well-informed decisions for patient care strategies that do not increase the risk of DED or exacerbate current symptoms of DED in their patients. A better understanding of the risk factors and recognition of signs and symptoms of DED may facilitate and expedite referrals to an eye care professional. Furthermore, we envision a wide-ranging partnership between health care providers across the health care spectrum and eye care professionals that diagnose and/or treat patients with DED that can foster a more interdisciplinary approach to recommending preventative measures and treatments.

## Definition and Pathophysiology of DED

Tears help to maintain the health of the ocular surface in addition to providing a smooth, refractive surface for optimal vision.^33,34^ Tears form a multilayered gradient fluid film over the ocular surface that helps to protect the cornea and lubricate blinking mechanics. The most superficial layer is a lipid layer that is deposited by small glands, called meibomian glands (MGs), lining the upper and lower eyelids during the blinking process and seals the tear film to reduce tear evaporation. The middle aqueous layer, composed of water, soluble mucins, and other proteins, constitutes 90% of the tear film volume and enables tear spreading^34,35^; this layer is secreted by the lacrimal and accessory lacrimal glands. The innermost mucous layer, made up of membrane-adherent mucins, interacts directly with the corneal epithelial cells.^34,35^

In healthy eyes, homeostasis of the tear film layers and their components is maintained within a very narrow, yet stable range.^34^ The homeostasis of the tear film, or loss thereof, can be assessed by measuring its osmolarity, which is more generically defined as the extent of solute particles in a solution.^36^ Tear film hyperosmolarity is, therefore, an increased amount of solute particles per tear film volume, and can result from reduced tear volume or increased tear evaporation.^33,34^

A central factor in the pathogenesis of DED is the loss of tear film stability, which usually results in hyperosmolarity, and leads to or may exacerbate damage to the ocular surface.^1,2,33,34^ Hyperosmolarity can result in symptoms of ocular discomfort, dryness, and/or vision disturbances. However, DED can also be present, as assessed by multiple clinical signs, in asymptomatic individuals. The chronic and sometimes progressive nature of DED is believed to be initiated by multiple factors that are self-perpetuated by an inflammatory cascade that causes further damage to the ocular surface.^33^ This, in turn, increases the inflammatory response and perpetuates a cycle of increased osmolarity and ocular surface damage. This “vicious circle” of DED can be complicated by a variety of lid and ocular surface diseases that further aggravate and damage the ocular surface.^13^

Hyperosmolarity of the tear film can result from reduced tear volume or increased tear evaporation.^33,34^ Aqueous deficient dry eye (ADDE) is defined by the presence of normal evaporation with a reduced tear volume and is the primary cause of DED in individuals with lacrimal gland dysfunction, such as in Sjögren's syndrome, rheumatoid arthritis (RA), diabetes mellitus, and age-related dry eye. Evaporative dry eye (EDE) is characterized by a normal tear volume with an increased rate of tear evaporation. EDE is the primary cause of DED in MG dysfunction and atrophy, and also is associated with Sjögren's syndrome.^1^ While there are two main types of DED defined, people with DED tend to fall within a spectrum of aqueous deficiency and evaporative disease, and often have signs of both present.^1^

## Epidemiology of DED

The epidemiology of DED has been increasingly studied since the publication of the 2007 Dry Eye Disease Workshop (DEWS) report,^2^ an encyclopedic review of the current state of DED knowledge and research. It was used to identify future research efforts needed to improve the understanding of the etiology and pathogenesis of DED to facilitate development of potential therapies. However, a central difficulty in these efforts has been establishing the criteria for diagnosis of DED.

Criteria for diagnosing DED vary considerably across epidemiological studies because of high variability in the clinical presentation and numerous causes and/or complicating factors of DED. A diagnosis of DED is based on self-reported signs and symptoms in response to standardized questionnaires on ocular discomfort (physical sensation, intensity, and frequency) and clinical tests (*e.g.*, corneal staining and measures of tear break-up time). For comparisons across epidemiological studies using similar or the same criteria for diagnosis of DED, and where the prevalence and incidence of DED in population-based studies has been stratified by gender and age, it has been consistently found that DED increases with age and disproportionately affects women.^3–7,9–11,37,38^

A meta-analysis of recent DED prevalence studies conducted by the 2017 TFOS DEWS II researchers found increases in the prevalence of DED signs and symptoms per decade starting at ages 40–49 years.^11^ For women, the prevalence of DED symptoms starts increasing from 14% at 50 years of age to 22% for those 80+ years of age. This trend is both less prominent and starts later for men, with a prevalence of DED symptoms increasing from 7% in men 60–69 years of age to 13% for those 80+ years of age.

Two large studies of DED prevalence conducted in the United States using the same criteria for DED diagnosis, the 2003 Women's Health Study (WHS; *n* = 39,000 females, ages 49+ years) and the 2009 Physicians' Health Study (PHS; *n* = 25,000 males, ages 50+ years), were used to draw comparisons of DED between genders and across ages.^6,12,39^ The studies found that the mean age of patients at diagnosis of their DED was 60 years for women versus 66 years for men. Ocular Surface Disease Index (OSDI)^40^ and the Symptom Assessment in Dry Eye (SANDE)^41^ are two of several validated dry eye questionnaires; evaluation using these questionnaires showed that severe symptoms were more common in women.^6^ Prevalence values from these studies were used to estimate DED prevalence in the United States population at nearly twice as high for women (3.25 million) as men (1.68 million).^6,12,39^

Table 1 summarizes recent DED studies from the United States that stratify DED prevalence and associated signs and symptoms by sex and/or gender. All of the studies generally support that major DED risk factors include female sex and age. The Beaver Dam Offspring Study (BOSS, 2014) found that prevalence in women was 17.9% versus 10.5% in men and that hormone therapy (HT) in women was an additional risk factor for DED.^7^

**Table 1.** Dry Eye Disease Prevalence From United States Studies

*Study*	*Type*	*Years conducted*	N	*Age range (years)*	*Female (%)*	*DED prevalence overall (%)*	*DED prevalence in females (%)*	*DED ratio F/M*
WHS^12^	Population	1992+	36,995	49–89	100.0	7.8	7.8	1.8^a^
PHS^39^	Population	1982+	25,444	50–99	0.0	4.3	N/A	1.8^a^
BOSS^7^	Population	2005–2008	3,285	21–84	54.6	14.5	17.9	1.7
NHWS^32^	Population	2013	75,000	18–49	50.4	8.8	4.5	2.0
DoD MHS^127^	Retrospective database analysis	2003–2015	9,732,272	2–90+	48.1	5.3	7.8	2.6

^a^ Ratio of female to male (F/M) refers to the Women's Health Study/Physician's Health Study (WHS/PHS).

BOSS, Beaver Dam Offspring Study; DED, dry eye disease; DoD, Department of Defense; MHS, Military Health System; N/A, not applicable; NHWS, National Health and Wellness Survey.

An analysis of data from the United States National Health and Wellness Survey (NHWS, 2017) found that gender differences in the prevalence of DED increased with age.^32^ While the prevalence of DED was nominally higher for women versus men in younger years (age 18–34 years: 2.9% vs. 2.6%), this difference increased significantly for older respondents (age ≥75 years: 22.8% vs. 12.6%).

Bradley et al. reported on a study of the United States Department of Defense (DoD) Military Health System (MHS) claims database analysis (2017), which covers over 9.7 million active military duty and retiree beneficiaries and their dependents from ages 2–80+ years.^42^ MHS data analysis revealed higher prevalence of DED in women (7.8% vs. 3.0% in men), and increases with age (18–39 years: 3.1% vs. 1.3%; ≥50 years: 15.9% vs. 7.0%).^42^

These differences in DED prevalence between women and men fall in the domain of gender effects on health. In addition to differences in metabolism, lifestyle, and physical performance, this rich field of study has identified sex differences in response to therapeutic agents, diagnostic and therapeutic interventions, sex differences in autoimmune conditions, and a spectrum of sex differences across common diseases such as coronary heart disease.^43^ DED prevalence differences are attributed to biological sex, hormonal, and gender differences, which contribute to DED risk factors, presentation, immune response, pain experience, and treatment response that are specific to women. Fortunately, there are differences in women's health care-seeking behaviors and health care service utilization, which will be discussed later, that provide opportunities for earlier referral and diagnosis of DED.^19^

## Biological Sex and Hormonal Differences in Ocular Structure, Functioning, and Health

Biological sex differences that affect ocular structure, functioning, and health are noted from the molecular level to the physiological observational level. Molecular-level differences include differences in tissue morphology, gene expression, protein synthesis, and epithelial cell dynamics.^10^ These molecular-level differences translate to differences in aqueous tear output, lipid production, mucous secretion, tear film stability, blink rate, and ocular immune functioning, which, when pathologic, may contribute to DED signs and symptoms.

For example, the transglutaminase 1 (TGase1) gene, which catalyzes protein crosslinking and thus plays a role in keratinization mechanisms, has greater expression in the healthy female cornea than the healthy male cornea by about 2:1.^44^ An *in vivo* vitamin A deficiency model of dry eye showed that TGase1 transcript and protein levels increased with time and disease severity.^45^ Pathologic keratinization of the cornea and conjunctival epithelium in patients with severe chronic dry eye, such as that associated with Sjögren's Syndrome and Stevens–Johnson syndrome, is accompanied by upregulation of TGase1.^46–48^ It has been hypothesized that the increased expression of TGase1 in women, when combined with differentiated levels of other important ocular sex steroids (androgens and estrogens), could be a contributing factor in the increased prevalence of DED in women.^44^

Hormonal differences between men and women, including both basal and lifespan-associated levels of sex steroids (androgens and estrogens), as well as hormonal cycles specific to the female sex (menstruation, pregnancy, menopause), also affect ocular structure, functioning, and health. Studies of associations of DED and sex steroid levels (testosterone, 4-androstene-3, 17-dione, estrogen, estrone, 17-β-estradiol, progesterone, and 17α-hydroxyprogesterone) have produced a variety of results regarding the levels of many sex steroids, especially estrogen, and their association with DED.^10,49–53^ Among these, low androgen levels are most consistently associated with DED.^51^ Sex steroids can be assessed systemically, by sampling of blood serum levels, or locally, by quantification of their presence in tears or ocular tissues.^53^ The associations of sex steroids to ocular diseases and DED have yet to be fully determined, but consensus holds that their influence on ocular surface conditions should be considered.

The association between DED and both systemic and ocular testosterone levels in women has yielded conflicting and/or inconclusive results. Blood serum levels of testosterone in postmenopausal women were assessed against corresponding OSDI scores in a small study between those with mild-to-moderate DED (*n* = 15), those with severe dry eye (*n* = 4), and women with no dry eye (*n* = 5),^49^ revealing no significant relationship between the two. In a study of 44 postmenopausal women, half with severe EDE (*n* = 22) and half without (*n* = 22), profiles of fasting blood serum levels of 17-β-estradiol, estrone, and total testosterone were compared with results from detailed eye examinations. The levels of 17-β-estradiol, estrone, and total testosterone in women with severe EDE showed inverse correlations to tear film osmolarity (regression coefficient, *r* = −0.7, −0.88, and −0.81, respectively).^50^

A method of steroid profiling in tear fluid was first published by Pieragostino et al. (simultaneous quantification of cortisol, corticosterone, 11-deoxycortisol, 4-androstene-3,17-dione, testosterone, 17α-hydroxyprogesterone, and progesterone levels).^53^ These steroids were extracted from tears collected on a Schirmer strip, followed by subsequent analysis by high-performance liquid chromatography/tandem mass spectrometry. This method was used in an analysis of tear samples from 14 females with DED and 13 healthy female controls, which found significant decreases in cortisol, 4-androstene-3,17-dione, and 17α-hydroxyprogesterone levels for patients with dry eye compared with controls.^53^

In a separate study, venous blood sampling and meibography were used to evaluate the association between serum levels of testosterone and estradiol and MG morphology in postmenopausal women (*n* = 198, average age 61.2 [±9.1] years).^52^ MG dropout increased with higher testosterone levels, and a significant difference in testosterone levels was observed in the mild versus severe MG dropout groups (MG dropout scores of 1 and 4, respectively; *p* = 0.002). Differences were not significant with estradiol and meibography assessments (*p* = 0.53).

Sex-associated differences in ocular physiology have been identified in the MG,^54^ lacrimal gland,^55^ conjunctiva,^56^ and cornea,^8^ as well as other eye structures (anterior chamber, ciliary body, iris, lens, retina, and vitreous). These physiological differences may contribute to the different DED prevalence rates observed in women and men.^10^ For example, sex-specific changes in the cornea occur during the menstrual cycle, pregnancy, and menopause, and include variations in thickness, hydration, curvature, sensitivity, and endothelial pigmentation, as well as contact lens tolerance and a higher prevalence of a loss of visual acuity.^8^

Some studies have suggested that changes in estrogen levels alter ocular surface equilibrium during the menstrual cycle, impacting subjective dry eye symptoms in female patients.^57^ In women with dry eye in particular, the estrogen peak (during the follicular phase) appears to be related to symptoms of impaired tear production and stability, surface dryness, and inflammation.^58^

Sex-related differences in the innate and adaptive immune responses of the ocular surface have been found to be minimal in the absence of systemic diseases.^59^ However, the close association of DED and autoimmune diseases that disproportionately affect women may also suggest that ocular surface immunity differences between the sexes manifest when the immune system is challenged.^10^

## DED-Associated Comorbidities Burden and Autoimmune Diseases Primarily Affecting Women

**FIG. 1.**
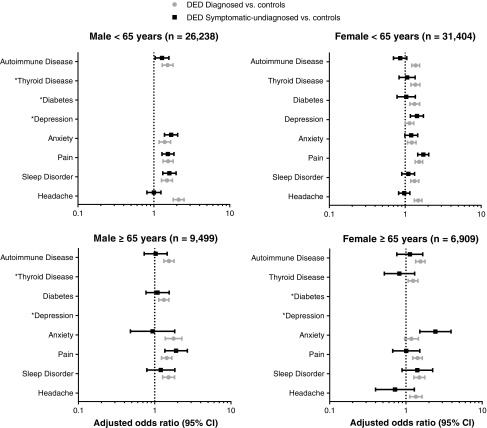
Risk of DED by comorbidities of interest from NHWS Study. No data are shown for comorbid conditions where there was no significant association with DED status in the strata of interest. Shire data on file. CI, confidence interval; DED, dry eye disease; NHWS, National Health and Wellness Survey.

DED is comorbid with a wide range of conditions. In the NHWS analysis, the extent of this association with various comorbidities differed between symptomatic and asymptomatic DED as well as with sex (Fig. 1, Shire data on file). All respondent women in the study with thyroid disease or depression were at greater risk of symptomatic DED compared with men. The analysis was further stratified by age groups. For survey respondents <65 years, diabetes mellitus was an additional risk factor for symptomatic DED in women but not men, and for those ≥65 years of age, headache/migraine or depression in the preceding 12 months were additional risk factors in women but not men. While headache, as a symptom, can be a very broad term—that is, not every patient with a headache is at risk for DED—older women who describe frequent or regular headaches/migraine that also have coexisting ocular symptoms may require a referral for possible DED.

In both age groups, men diagnosed with DED were at a higher risk of having glaucoma than women with any type of DED. Anxiety was a much higher risk for symptomatic DED in women ≥65 years of age than for men. In the analysis of the WHS versus PHS studies,^6^ there was a significantly higher comorbid rate of Sjögren's syndrome, systemic lupus erythematosus (SLE), and rosacea in women. For men, higher comorbid rates of blepharitis and MG dysfunction were observed.

Autoimmune diseases are generally more prevalent in women than in men.^60^ Severe dry eye can be precipitated by several common autoimmune conditions in which inflammation plays a key role: Sjögren's syndrome, SLE, RA, and thyroid diseases.^60^ Although the link between rosacea and autoimmunity is still under debate, rosacea is a chronic inflammatory disease that is often associated with DED.^25,61,62^ In these diseases, females represent the majority of cases (ratio of women:men)^63^; Sjögren's syndrome (9:1), SLE (7:1), RA (3:1), thyroid diseases (3.1–5:1), and rosacea (3:1) (Table 2).

**Table 2.** Autoimmune Diseases Associated with Dry Eye Disease^63^

*Autoimmune disease*	*Ratio women:men*	*DED association (DED type/manifestation)*
Rosacea	3:1	Chronic, cutaneous inflammatory disease with ocular symptoms, including foreign body sensation, burning, irritation, tearing, photophobia, blurred vision, and red eye
		Contributes to meibomian gland inflammation, dysfunction, and EDE
Rheumatoid arthritis	3:1	Ophthalmic manifestation of chronic inflammation—keratitis, MGD (ADDE+EDE)
Hashimoto's thyroiditis; Graves' disease	3–5:1; 7:1	T lymphocytes and autoantibodies directed against specific orbital or thyroid-and-orbital shared antigen(s) causing thyroid eye disease (EDE)
SLE	7:1	Immune complex deposition in the lacrimal gland caused by SLE may result in secondary Sjögren's syndrome causing dry eye due to lack of adequate tear production (ADDE)
Sjögren's syndrome	9:1	Dry eye due to lack of adequate tear production and chronic inflammation may lead to MGD (ADDE+EDE)

ADDE, aqueous deficient dry eye; EDE, evaporative dry eye; MGD, meibomian gland disease; SLE, systemic lupus erythematosus.

In Sjögren's syndrome, a chronic inflammatory disorder, lymphocytic infiltration of the lacrimal glands precipitates ADDE.^21,64^ In the lacrimal gland tissue of women with Sjögren's syndrome, androgen deficiency is both a sign and potentially a cause of the local steroidal imbalance precipitating lacrimal gland inflammation and ADDE.^65^ Further research is needed to understand the specific lacrimal and ocular surface mechanisms of EDE in relation to Sjögren's syndrome.

SLE is a chronic, multisystem, autoimmune disease, and DED is its most common ocular manifestation.^25^ Ocular symptoms can correlate with systemic disease progression, which may present before other systemic signs of SLE, underscoring the importance of ophthalmological examinations for SLE patients.^66^ Immune complex deposition in the lacrimal gland caused by SLE may result in secondary Sjögren's syndrome causing dry eye due to lack of adequate tear production (ADDE). Approximately 20% of SLE patients experience coexisting Sjögren's syndrome.^67^

RA is a chronic autoimmune inflammatory polyarthritis that causes irreversible synovial joint damage.^25^ ADDE is the most common ophthalmic manifestation of RA.^68^ DED prevalence in RA patients is reported to be 15%–25%.^69^ DED in RA patients can be caused by the inflammatory effects of the disease, but DED may also manifest iatrogenically through treatment of the arthritis with corticosteroids and hydroxychloroquine.^29^ New categories of disease-modifying antirheumatic drugs that block the Janus kinase pathways in the immune response are available; however, their effect on ocular immune response, and their risk for development of DED, is not yet known.^70,71^

It is common for patients with rosacea to have ocular signs and symptoms. These can include blurred vision, photophobia, tearing, burning, irritation, foreign body sensation, and red eyes.^25^ Inflammation of the tissue around the MGs and subsequent alteration to the lipids produced by the glands is hypothesized to be a cause of the ocular manifestations in these patients and can lead to tear film abnormalities and EDE. Ocular rosacea also causes a reduction in tear volume in more than one-third of those with the ocular manifestation.^72^ A study was undertaken to compare the symptomatic relief from ocular complications of rosacea that could be obtained from treatment with topical cyclosporine ophthalmic emulsion 0.05% versus oral doxycycline, the reference treatment for ocular rosacea. The topical ophthalmic treatment was found to be more effective than the systemic medication more commonly prescribed for rosacea patients.^73,74^

Autoimmune thyroid eye disease, such as Hashimoto's thyroiditis or Graves' disease, is a disorder in which the ocular tissues are attacked and become inflamed.^75^ Women are five to six times more likely than men to get thyroid eye disease.^76^ It is believed that thyroid antibodies target eye muscles and connective tissue within the eye socket because these tissues contain proteins that appear similar to those of the thyroid gland. Dry eye and irritation in these patients are attributable to inflammation and swelling of tissue around the eye that causes incomplete blinking and an inability to shut the eyes. Thyroid eye disease must be treated as a separate ocular condition to the thyroid imbalance since systemic treatment of the thyroid gland does not consistently improve thyroid eye disease.^76^

## DED and Chronic Pain

Associations have also been found between DED and chronic pain.^18,23,77–80^ Like DED, risk factors for many chronic pain syndromes (CPS) are female sex and older age.^81,82^ The higher incidence of pain-related symptoms among women with DED compared with men with DED suggests an underlying biological mechanism that may be sex related.^10^

In the Netherlands, a study of 425 patients with DED found that 17% of DED patients had at least one other CPS, such as chronic pelvic pain, fibromyalgia, or irritable bowel syndrome.^82^ When assessed by the OSDI symptom survey, DED patients with CPS had a mean OSDI score of 45.8, versus 33.8 in DED patients with no CPS. OSDI score is on a scale of 0–100; normal range is 0–10, clinical sensitivity for DED begins at an OSDI score of ≥13, moderate DED 23–32, and severe DED ≥33.^40,83^ Of 64 patients from the United Kingdom Twins Registry who were diagnosed with DED, 38% (24/64) also had CPS and higher OSDI symptom scores compared with those with no CPS (OSDI score 34.1 vs. 14.4; *p* = 0.001).^84^

From a prospective, cross-sectional study of 40 DED patients with no other known systemic disease, age and gender matched to 20 patients with fibromyalgia and 20 healthy controls,^85^ it was found that 62% of patients with fibromyalgia had DED based on OSDI scores. Ocular and visual symptoms self-reported from the OSDI and the National Eye Institute Visual Functioning Questionnaire (VFQ-25) were statistically higher in DED and fibromyalgia groups when compared with the control group (*p* < 0.0001).

Vehof and researchers from the Netherlands and the United Kingdom have hypothesized that the chronic pain in DED may be a neuropathic type of pain, persisting through nociceptive pathways.^82^ Clinical consequences of corneal damage activate an alarm system integrated in the peripheral and central trigeminal sensory network. Pathological elements in this response lead to a neuralgia that is characterized by a large disparity between intense pain symptoms and clinical signs correlating to those symptoms.^79^ They also found the severity of signs of DED had a lower correlation to symptoms in women compared with men (*ρ =* 0.11 in women vs. *ρ* = 0.33 in men; *p* = 0.01).^86^ The lack of concordance between symptoms and signs of DED has contributed to the lack of standardized diagnosis used across clinical studies.^77,87,88^

## Iatrogenic and Gender Differences That Increase DED Risk in Women

We must also consider iatrogenic causes of DED and cultural behaviors specific to women that may increase the risks of DED. Iatrogenic causes include contact lens use; elective ophthalmic procedures, such as laser-assisted *in situ* keratomileusis (LASIK) for refractive error correction; periorbital surgeries to improve blinking mechanics or for cosmesis; and both systemic and topical medical treatments, such as hormone replacement therapies and allergy eye drops.^59^ Behavioral causes of DED include cosmetic periorbital surgeries and the use of permanent and topical cosmetics and facial creams.^59^ Some of the external causes of DED are listed in Table 3.

**Table 3.** Iatrogenic Procedures and Dry Eye Disease

*Iatrogenic and elective causes of DED*	*Reason for procedure or use*	*DED causation*
Blepharoplasty	Therapeutic or cosmetic eyelid surgery	Incomplete blinking and exposure of ocular surface^99,101,128^
Laser *in situ* keratomileusis (LASIK)	Refractive correction	Alteration of corneal shape and/or neural feedback loop dysregulation^59,129–131^
Botulinum toxin type A injections (BTX-A)	Therapeutic or cosmetic site-specific muscle block	Incomplete blinking and exposure of ocular surface^59,104^
Contact lenses	Vision correction	Reduced oxygen and increased friction to ocular surface^7,72,112,132^
Prescribed medications	Oral and ophthalmic topical, therapeutic for other conditions	Secondary dryness effects related to medication use, preservatives in ophthalmic topical treatments^5,7,17,59,107,133–135^
Permanent eye cosmetics (tattoos)	Cosmetic	Destruction of meibomian glands^59,105^
Topical cosmetics and facial creams	Cosmetic and protective	Increased debris on corneal surface, retinoids and oils from periorbital application of creams may cause meibomian gland atrophy^59,133^

Two-thirds of the more than 45 million contact lens users in the United States are women.^89,90^ In some individuals, depending on the type of contact lens, long-term contact lens wear has been found to desensitize the cornea.^91^ In a large epidemiological study in Canada, contact lens use was found to increase the risk of experiencing DED symptoms.^92^ In a questionnaire-based study of contact lens wearers in the United States, ocular surface discomfort (scratchiness) was significantly higher for women than for men (*p* < 0.008).^93^ In addition, dryness and discomfort are the leading causes of contact lens discontinuation.^94,95^ In a recent study, contact lens use was found to accelerate age-related morphological changes in MGs, such as MG dropout, which may be associated with DED.^96^

LASIK is a procedure performed for the surgical correction of refractive error. In the United States, LASIK has been performed in over 21 million patients as of 2015.^97^ LASIK may increase the risk for DED, particularly in women.^97,98^ Data from the UK Biobank Study found that LASIK procedures were performed more often in women than in men, although the incidence of this procedure and the gender difference decreased with age.^97^ A 1-year retrospective analysis of 88 patients who underwent LASIK for treatment of hyperopia concluded that DED was particularly problematic in females and was associated with refractive regression.^98^

Blepharoplasty after a LASIK procedure creates an increased risk for development of severe DED.^59^ This is because the LASIK procedure can reduce corneal sensitivity, resulting in a decreased blink rate and subsequent reduction in tear production. Postprocedure, blepharoplasty causes transient (or infrequently, permanent) lagophthalmos, which is compensated for by an increase in blink rate. However, if the tear volume is insufficient because of a prior LASIK procedure, the increased blink rate can cause mechanical irritation.^99^

In the United States in 2015, women accounted for 85% of those presenting for more than 200,000 cosmetic blepharoplasty procedures.^100^ From a 2013 10-year retrospective medical record review of patients undergoing cosmetic blepharoplasty (892 patients, 16–83 years of age with a mean age of 53 years), 86.1% were female and 63% underwent concurrent upper and lower blepharoplasty. Compared with an upper or lower blepharoplasty alone, the concurrent procedure was more highly associated with the percentage of patients that subsequently developed DED (31.3% concurrent vs. 12.9% upper and 22.9% lower, chi square test = 18.1, *p* < 0.001).^101^

Botulinum toxin type A (BTX-A) injections^102^ are used in ophthalmology to improve a number of conditions, including DED. BTX-A injections into the periorbital area, such as the orbicularis muscle, have been used successfully to treat dry eye by reducing lacrimal drainage.^103^ However, repeated BTX-A injections into lateral canthal rhytids for cosmetic effect, more commonly known as correction of crow's feet, may cause temporary dry eye due to muscle weakness that results in incomplete blinking.^104^ As the effect of these injections is transient, the experience of dry eye symptoms may also be transient. However, if DED is already present, or the patient has comorbidities that incur a risk for DED, the transient cause may lead to a chronic condition.^102–104^

Dermapigmentation of the eyelids, or cosmetic eye tattooing, can cause a destruction of the MGs due to excessive needle penetration and ink pigment migration.^105^ Loss of MGs in these cases reduces the lipid layer of the tear film and can lead to EDE. Topical ocular cosmetics, especially mascara and eyeliner, as well as oil-based facial creams applied near the eye, can cause changes to the tear film and its stability. Retinoids in moisturizing creams have been associated with problems in MGs.^10^ In a study that compared ocular comfort and OSDI scores between cosmetic users (*N* = 1,360 females, median age 25 years; 83% reported wearing cosmetics >3 times per week [mascara most common]) and nonusers, OSDI scores were found to be similar (*p* = 0.083)^106^ When cosmetics were not used by habitual users, the perception of ocular comfort increased (*p* < 0.001).

Prescribed medications can also contribute to the development of dry eye.^59^ These can arise from side effects, which cause systemic changes that decrease secretory fluids and result in dryness of mucous membranes (*e.g.*, dry mouth, dry eyes), or induce local changes in eye function (*e.g.*, reduction in production of tear film components, induction of inflammation, and/or swelling of ocular tissues).^59^ Active ingredients or inactive ingredients (*i.e.*, preservatives) of topical ophthalmic medications may cause or aggravate eye dryness, irritating ocular tissues or destabilizing the tear film.

While preservative-free preparations of ophthalmic products are commonly available, many ophthalmic preparations contain preservatives to prevent microbial proliferation, deciphering the contribution of the active versus inactive ingredient as it relates to DED can be difficult. The beta blocker timolol induces tear film instability and disrupts corneal barrier function, and the antihistamine olopatadine contributes to reduced tear volume.^108^ Preservatives that may contribute to dry eye symptoms include benzalkonium chloride (BAK); benzethonium chloride and cetyl pyridinium chloride; benzyl bromide; benzyl, cetyl, and phenylethyl alcohols; chlorobutanol; edetate disodium (EDTA); phenylmercury nitrate, acetate, and borate; thimerosal; merthiolate; polymyxin B sulfate; chlorhexidine; methyl and propyl parabens; quaternary ammonium chloride; sodium benzoate and propionate; and sorbic acid.^109^ The most widely used ocular administration preservative, BAK, has been shown to have cytotoxic and proinflammatory effects on the eye, and its detergent properties disrupt the tear film.^59^

In the Beaver Dam Eye Study cohort, previous use of daily over-the-counter medications, including aspirin (odds ratio 1.18 [95% confidence interval 0.99–1.40] *p* = 0.06) and/or multivitamins (1.37 [1.08–1.74]; *p* < 0.005), were nearly or statistically significantly associated with age- and sex-adjusted prevalence of dry eye.^3^ Among the more than dozen classes of medications identified that provide increased risks of DED, several of these classes are specific to women (*e.g.*, hormone replacement therapy), and/or are used significantly more by women than by men across age groups (antihistamines, anxiolytics, antidepressants, antipsychotics).^6,59,107,110^ Examples of specific systemic medications associated with DED are listed in Table 4.

**Table 4.** Selected and Illustrative Systemic Medications Associated with Dry Eye Disease (adapted^135^)

*Medication type*	*Medications with reported side effect categories, including burning sensation, keratoconjunctivitis sicca, decreased lacrimation, meibomian gland changes, sicca-ocular, Stevens–Johnson syndrome*
Antidepressants	Agomelatine, Amitriptyline, Bupropion, Clomipramine, Citalopram, Desipramine, Doxepin, Duloxetine, Fluoxetine, Fluvoxamine, Imipramine, Mianserin, Mirtazapine, Nortriptyline, Paroxetine, Reboxetine, Sertraline, Tianeptine, Trazodone, Venlafaxine
Antihistamines	Azelastine, Brompheniramine^*^, Carbinoxamine^*^, Cetirizine^*^, Chlorpheniramine^*^, Clemastine^*^, Cyproheptadine^*^, Desloratadine^*^, Dexchlorpheniramine^*^, Diphenhydramine^*^, Doxylamine^*^, Epinastine, Fexofenadine^*^, Hydroxyzine, Ketotifen, Loratadine^*^, Olopatadine, Promethazine, Pseudoephedrine, Tripelennamine^*^, Triprolidine^*^
Antipsychotics	Aiprasidone, Aripiprazole, Brompheniramine, Carbinoxamine, Chlorpheniramine, Chlorpromazine, Clemastine, Clozapine, Cyproheptadine, Dexchlorpheniramine, Fluphenazine, Haloperidol, Lithium carbonate, Olanzapine, Perphenazine, Promethazine, Quetiapine, Risperidone, Sulpiride, Thiethylperazine, Thioridazine Thiothixene, Trifluoperazine
Anxiolytics	Alprazolam^*^, Diazepam^*^, Eszopiclone, Lorazepam^*^, Zolpidem, Zopiclone
Hormonal	Alfuzosin, Doxazosin, Finasteride^*^, Leuprorelin^*^, Tamsulosin, Terazosin, Estrogen/progesterone, Medroxyprogesterone
Neurotoxins	Botulinum A^*^ or B

“Associated” specifically refers to systemic medications causing, contributing to, or aggravating dry eye.

^*^ Drugs identified with a causative relationship to dry eye symptoms as described by Fraunfelder et al.^135^

The association of DED and hormone treatments in women is not well understood but may involve adverse effects of increased ocular levels of estrogen on MGs or production of tear film components. In the WHS analysis, postmenopausal HT increased DED risk by ∼70% for estrogen users and ∼30% for estrogen+progesterone/progestin users.^12^ A cross-sectional study of 360 postmenopausal women investigating the effect of HT on DED showed significant differences in increased DED severity between the following groups: HT versus no HT (*p* < 0.0001); HT dosage level >1 mg/day versus <1 mg/day (*p* < 0.0001); and HT duration of at least 12, 36, or 48 months (*p* < 0.0001).^111^ Oral contraceptive pill (OCP) use in premenopausal women (*n* = 52 OCP, *n* = 45 non-OCP, mean age, 26.0 ± 3.7 years), resulted in higher SANDE scores (*p* < 0.01) and the combination of OCP and recent contact lens wear were associated with higher SANDE (*p* < 0.001) and OSDI scores (*p* = 0.015).^112^

Serotonin/norepinephrine reuptake inhibitors (SNRIs), used to treat depression, have known anticholinergic adverse effects, including decreased lacrimal secretion, that increase the risk of dry eye.^110^ Selective serotonin reuptake inhibitors (SSRIs), an alternative class of medication for treatment of depression, do not adversely affect the cholinergic system.

However, in an investigation of tear film volume (classified as >5 or ≤5 mm by the Schirmer test) with antidepressant use (≥4 weeks), duloxetine (SNRI), venlafaxine (SNRI), and escitalopram (SSRI) significantly reduced tear film volume (*p* < 0.001) compared with control patients not on antidepressants. This finding suggests that SSRIs may impact tear film volume through a mechanism outside the cholinergic system. Patients using SSRIs had lower wetting measurements compared with those on SNRIs (*p* < 0.05). However, both SNRIs and SSRIs may contribute to dry eye.^110^ In the SSRI group, 10 patients (19.8% of 54 eyes) had dry eye, and in the SNRI group 19 patients (35% of 54 eyes) had dry eye. The tricyclic antidepressants amitriptyline and thioridazine for schizophrenia also have anticholinergic effects that increase the risk for DED.^113^

## Gender Differences in Health-Related Quality of Life

Indicators associated with overall physical and mental health include adequate sleep; stable moods; balanced nutrition; and minimal stress, anxiety, and depression.^32,114–117^ For many of these indicators, women fare poorer in self-reports than men,^19^ and among DED patients women report worse health-related quality of life and perceived health than men.^6,9,21^ As compared with men, women report a greater impact of DED on visual quality indicators, including poor vision, blurred vision, and fluctuating/unstable vision, as well as sustained visual attention tasks, including reading, night driving, television viewing, and computer/monitor work.^6^

A study of 890 women 30–69 years of age found that Pittsburgh Sleep Quality Index scores (mean ± SD) were worse in patients with DED (6.1 ± 2.9) versus without DED (4.9 ± 2.7); *p* = 0.003.^17^ The Hospital Anxiety and Depression Scores (mean ± SD) in the same study were worse in patients with DED 30–45 years of age (13.2 ± 6.0) than in those without DED (9.7 ± 6.0); *p* = 0.02.^17^

A 2010–2011 analysis of the Korea National Health and Nutrition Examination Survey (a population-based cross-sectional study) in 6,655 females was carried out to look at the association between DED signs and symptoms and depressive symptoms.^16^ Across all respondents, 20% experienced DED symptoms, and DED prevalence from a confirmed diagnosis was 12.3%. Also, a DED diagnosis was associated with greater odds of experiencing anxiety and/or depression (odds ratio [OR], 1.5; 95% CI: 1.1–2.0), depressive mood (OR 1.5; 95% CI: 1.1–2.0), severe psychological stress (OR 2.5; 95% CI: 1.6–4.0), and of having had prior psychological counseling (OR 1.8; 95% CI: 1.0–3.1).

## Gender Differences in Care-seeking Behaviors and Health Care Service Utilization

Women have been found to interact more and differently with the health care system than men^118^ in ways that offer opportunities for earlier detection, referral, preventative treatment, and/or diagnosis in the treatment of DED. Based on a survey of 1,518 women and 581 men who previously participated in the WHS^12^ and PHS I and II,^39^ more women than men reported using traditional DED therapies (level 1).^6^ These included artificial tears (82.8% vs. 62.6%; *p* < 0.0001), lubricating eye ointments (19.2% vs. 11.7%; *p* < 0.0001), and hot compresses (14.3% vs. 10.7%; *p* < 0.02).^6^ This is also true of the next tier of DED therapies (level 2); omega-3 fatty acids (18.6% vs. 9.6%; *p* = 0.0006), cyclosporine eye drops (13.4% vs. 6.4%; *p* < 0.0001), and punctal occlusion (15.0% vs. 9.1%; *p* = 0.005).^6^

Overall, the same level of general satisfaction with DED treatments was reported, however, compared with men, women reported greater dissatisfaction with side effects of DED therapies and the amount of time for the treatment to alleviate symptoms. In an alternate model, dissatisfaction with the convenience of treatment use as instructed was less in women than in men (OR 0.74; 95% CI: 0.58–0.95).^6^

Overall, compared with men, women may try a more diverse set of treatments, including nutritional supplements and other over-the-counter therapies.^119,120^ Tear film homeostasis has been found to be dependent on adequate nutrition, particularly sufficient intake of protein and vitamin A.^121^ A low-fat diet, rich in antioxidants and omega fatty acids, has previously been shown to benefit both the tear film and ocular surface,^122,123^ leading many clinicians to recommend omega-3 fatty acids to patients to alleviate DED symptoms.

Schaumberg et al. found that women use omega-3 fatty acids for the treatment of their DED symptoms significantly more often than men.^6^ A 2013 multicenter double-masked placebo-controlled clinical trial of 38 postmenopausal women with moderate-to-severe DED was conducted to measure the effect of supplementation with gamma-linolenic acid (GLA) and omega-3 (n-3) polyunsaturated fatty acids (PUFAs) on DED signs and symptoms.^124^ A change from baseline to 24 weeks in OSDI scores of women taking supplements (*N* = 19) were significantly improved compared with women taking placebo (*N* = 19); OSDI score 21 ± 4 vs. 34 ± 5; *p* = 0.05. Also at 24 weeks, conjunctival impression cytology measures of CD11c integrin and HLA-DR expression, both DED disease-relevant inflammatory mediators, showed increases of >30% for placebo compared with supplement treatment (*p* = 0.001). However, no differences between placebo and supplement treatment cohorts were found for tear production, tear breakup time, or corneal or conjunctival staining.^124^

In the recently published Dry Eye Assessment and Management (DREAM) study, a large randomized clinical trial, 535 DED patients with moderate-to-severe DED were assigned 2:1 to receive “active” or placebo supplements over 12 months.^125^ The active supplements contained 400 mg of eicosapentaenoic acid (EPA) and 200 mg of docosahexaenoic acid (DHA), both omega-3 (n-3) fatty acids, taken five times daily, for a total dose of 3,000 mg. Placebo capsules contained 1,000 mg of refined olive oil made up of 68% oleic acid (n-9), 13% palmitic acid (saturated), and 11% linoleic acid (n-6). Both active and placebo capsules contained an antioxidant; 3 mg of vitamin E (alpha-tocopherol). During the trial, patients were allowed to continue their current treatments for DED. While the data in this study were not stratified by sex/gender or age, females comprised >80% of the omega-3 and placebo cohorts and the mean age was 58 years.^125^

After 12 months of treatment, signs and symptoms of DED improved significantly in both the active and placebo cohorts, with 53% of the active cohort and 57% of the placebo cohort reporting less use of additional treatments for their dry eye. Mean OSDI scores decreased by 13.9 ± 15.6 points for the active group and by 12.5 ± 18.2 points for the placebo group; however, the difference between groups was not significant (*p* = 0.40). Based on between-group differences in changes from baseline to 12 months in OSDI score, corneal staining, tear break-up time, and Schirmer's test, the omega-3 supplement did not show additional benefit compared with placebo. The olive oil placebo capsules used in this study, which predominantly contained oleic acid, an omega-9 PUFA, was not expected to have an effect on the signs and symptoms of DED.^125^

Methodological difficulties of the placebo effect in DED clinical trials of topical treatments have been previously reported.^126^ A combined analysis of the placebo arms of three clinical trials of diquafosol treatment for DED carried out in Japan, comprised of 205 DED patients (82.4% female with a mean ± SD age of 54.4 ± 17.9) found high baseline scores, indicating increased DED severity, and aging to be predictive of the magnitude of placebo response.^126^

## Conclusions

DED can be a complex and time-consuming condition to manage, and some individuals with DED experience refractory symptoms. It is well supported that the sex and gender differences between women and men, especially as they age, contribute to differences in the prevalence and severity of DED.

In this study, we have reviewed sex and gender differences in DED causes, clinical sign presentation, patient experience of symptoms, and—perhaps most relevantly—differences in treatment response and efficacy. While some of these differences are biological, with sex-specific hormones having a great effect on DED etiology, there are also comorbidities and gender-based factors that contribute to DED risk in women. Because there is a strong association of autoimmune diseases and DED, especially those that primarily affect women, both systemic and overall effects of DED need to be assessed in treatment options of DED and comorbidities. The high burden of comorbidities women with DED face suggests they would benefit from an interdisciplinary approach to DED treatment. Women are diagnosed with DED at earlier ages, and progression to severe forms of the disease is more prevalent in women than men. Thus, earlier diagnosis of DED in women may result in a significant improvement in their quality of life.

In light of the many women's health topics raised here with associations to DED, opportunities for educating women and health care providers on this highly prevalent and burdensome disease should be maximized; and when dry eye is suspected, referral to an optometrist or ophthalmologist for a dry eye examination is warranted.
